# Mobilizing faith-based COVID-19 health ambassadors to address COVID-19 health disparities among African American older adults in under-resourced communities: A hybrid, community-based participatory intervention

**DOI:** 10.1371/journal.pone.0285963

**Published:** 2024-02-15

**Authors:** Edward K. Adinkrah, Shahrzad Bazargan, Sharon Cobb, Lucy W. Kibe, Roberto Vargas, Joe Waller, Humberto Sanchez, Mohsen Bazargan

**Affiliations:** 1 Department of Family Medicine, Charles R. Drew University of Medicine and Science, Los Angeles, California, United States of America; 2 Departments of Psychiatry, Charles R. Drew University of Medicine and Science, Los Angeles, California, United States of America; 3 Department of Psychiatry and Biobehavioral Sciences, University of California Los Angeles, Los Angeles, California, United States of America; 4 Mervyn M. Dymally College of Nursing, Charles R. Drew University of Medicine and Science, Los Angeles, California, United States of America; 5 Physician Associate Program, Charles R. Drew University of Medicine and Science, Los Angeles, California, United States of America; 6 Department of Internal Medicine, Charles R. Drew University of Medicine and Science, Los Angeles, California, United States of America; 7 Office of Research, Charles R. Drew University of Medicine and Science, Los Angeles, California, United States of America; 8 Department of Family Medicine, University of California Los Angeles, Los Angeles, California, United States of America; Public Library of Science, UNITED KINGDOM

## Abstract

**Introduction:**

The COVID-19 pandemic disproportionately affected older adults, particularly those with pre-existing chronic health conditions. To address the health disparity and challenges faced by under-resourced African American older adults in South Los Angeles during this period, we implemented a hybrid (virtual/in-person), pre-post, community-based participatory intervention research project utilizing a faith-based lay health advisor model (COVID-19 Health Ambassador Program (CHAP)). We recruited COVID-19 Health Ambassadors (CHAs) and African American older adults (participants) from faith-based organizations who partook in CHA-led meetings and follow-ups that educated and supported the participants. This paper seeks to evaluate this intervention’s implementation using the Consolidated Framework for Implementation Research (CFIR) as a reporting tool with an emphasis on fidelity, challenges, and adaptations based on data collected via stakeholder interviews and surveys.

**Results:**

CHAP was delivered to 152 participants by 19 CHAs from 17 faith-based organizations. CHAs assisted with chronic disease management, resolved medication-related challenges, encouraged COVID-19 vaccination, reduced psychological stress and addressed healthcare avoidance behaviors such as COVID-19 vaccine hesitancy among the participants. Challenges encountered include ensuring participant engagement and retention in the virtual format and addressing technological barriers for CHAs and participants. Adaptations made to better suit the needs of participants included providing communication tools and additional training to CHAs to improve their proficiency in using virtual platforms in addition to adapting scientific/educational materials to suit our participants’ diverse cultural and linguistic needs.

**Conclusion:**

The community-centered hybrid approach in addition to our partnership with faith-based organizations and their respective COVID-19 health ambassadors proved to be essential in assisting underserved African American older adults manage chronic health conditions and address community-wide health disparities during the COVID-19 pandemic. Adaptability, cultural sensitivity, and teamwork are key to implementing health interventions especially in underserved populations.

## Introduction

African American (AA) older adults in South Los Angeles (LA) face peculiar healthcare challenges that transcend socioeconomic barriers. Representing 9% of the LA County population, many AA communities have historically been under-resourced due to a complex interplay of factors [1]. For instance, historic practices such as redlining have confined AA communities to specific neighborhoods, impeding economic and developmental growth [2]. This has had lingering repercussions. For instance, in 2019, the life expectancy for AA residents stood at 72 years, contrasting sharply with the 82 years for the county’s White population [3]. Such disparities, deeply rooted in structural racism, restricted access to health resources, and heightened predisposition to chronic health conditions further underline their challenges [4, 5]. With this historical backdrop, the onset of the COVID-19 pandemic introduced another layer of complexity to an already fragile health landscape [6–8]. Physical distancing requirements, as a countermeasure to the pandemic, significantly hampered community-partnered interventions, especially in AA communities that have traditionally leaned heavily on in-person gatherings for support and dissemination of vital health information. The pandemic not only disrupted the management of preexisting chronic health conditions but also limited this community’s access to primary and specialty healthcare providers and pharmacies. Further complicating matters was the struggle to access reliable information about COVID-19 prevention, testing, and vaccines [8–10]. Numerous studies have attempted to address these disparities; however, gaps in research and interventions persist, highlighting the need for innovative, community-centered solutions to promote health equity during the pandemic [11–13].

In response, we developed the COVID-19 Health Ambassador Program (CHAP), a community-partnered participatory initiative. It sought to bridge this gap by leveraging on faith-based organizations (FBOs) and the invaluable services of Lay Health Advisors (LHAs) in a hybrid (in-person and virtual) format. At its core, CHAP leveraged the traditional reliance of AA communities on faith-based organizations (FBOs), specifically, AA churches, who have been a trusted source of social support, advocacy, and resources [14, 15]. These organizations often have served as a key platform for disseminating health information, engaging in health-related screening and outreach efforts, and organizing educational workshops, health fairs, and seminars [16, 17]. In tandem with FBOs, Lay Health Advisors (LHAs) have served as crucial bridge-builders between communities and healthcare systems. These trained individuals, echoing the cultural, linguistic, and socioeconomic backgrounds of the communities they serve, have been paramount in fostering trust and delivering culturally sensitive information [18, 19]. The impact of LHAs as peer support and counselors, care coordinators, community advocates, and mobilizers is well established in research. Existing scientific evidence also supports the integration of LHAs from community churches into health interventions. This integration has been effective in achieving optimal health outcomes for AA older adults with chronic health conditions [20–22].

This paper’s main objective is to evaluate CHAP’s implementation and assess its impact on addressing health disparities among under-resourced older AA adults during the COVID-19 pandemic. This evaluation will be based on the Consolidated Framework for Implementation Research (CFIR), an established framework used to guide the systematic assessment of health intervention implementation [23, 24].

## Methods and materials

### Study design

This study utilized a mixed-methods, community-based participatory research (CBPR) approach to implement a “one group, pretest–posttest” intervention over two years. The combination of quantitative and qualitative research methods aimed to capture both the measurable impact and the nuanced experiences of the participants. The surveys focused on tracking shifts in participants’ knowledge, behaviors, and attitudes towards both COVID-19 and the broader realm of chronic disease management [25]. In tandem, qualitative tools, namely informal and semi-structured group/individual interviews, offered a window into the lived experiences, perceived benefits, and challenges encountered by participants during the study’s course [26, 27]. The adoption of CBPR was influenced by its capacity to enhance trust within underserved communities [28, 29]. Historically, certain research paradigms might have inadvertently reduced trust in such populations due to their prescriptive nature or misalignment with community-specific needs [30]. In the backdrop of the COVID-19 pandemic, characterized by misinformation and varying levels of trust in health interventions [31], CBPR, with its emphasis on mutual respect, collaborative learning, and collective decision-making, offered a potential solution to these challenges [28, 32, 33]. The study’s design leaned on the "one group pretest–posttest" framework given the disproportionate impact of COVID-19 on the African American community, where all participants in the study were inherently deemed high-risk in the context of COVID-19 susceptibility and the potential for adverse health consequences stemming from the disease [34]. Given this profound impact, it was an ethical imperative to ensure no high-risk individual was denied intervention. Furthermore, introducing a non-equivalent or quasi-experimental design would introduce not just methodological, but also ethical complications.

### Setting

**Fig 1:**
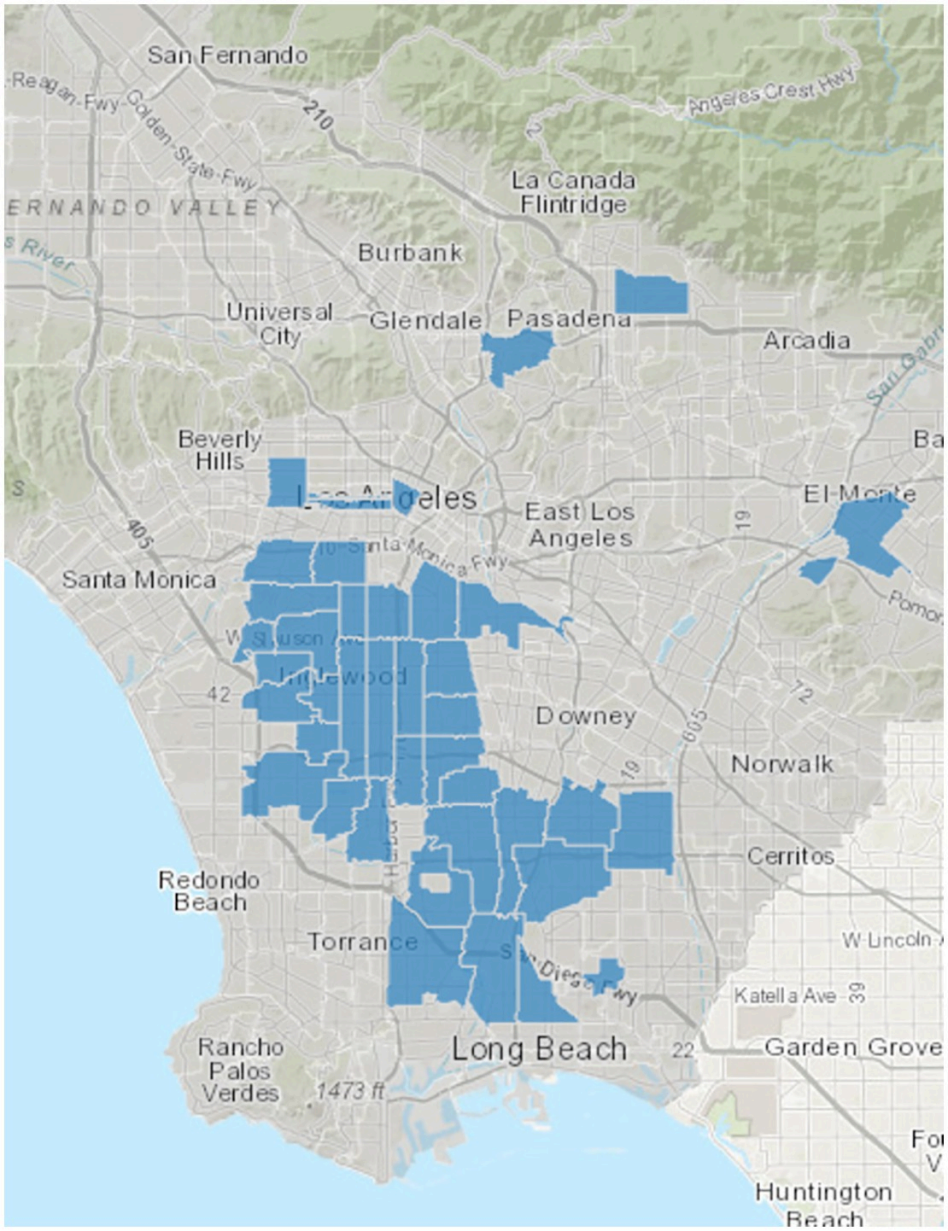
Map of the study setting highlighting areas representing the distribution of study participants.

This study was conducted in 17 faith-based organizations (FBOs) located primarily within urban regions of Los Angeles County’s Service Planning Area (SPA) 6, a region heavily impacted by the COVID-19 pandemic. Over 90% of these FBOs’ membership comprised African Americans. In comparison to the rest of Los Angeles County, individuals in SPA 6 face a higher prevalence of health challenges at a disproportionate rate [35]. While the diversity of faiths (such as Islam and Hinduism) in the study setting is noteworthy, our research exclusively engaged Christian-based FBOs. The FBOs, located within a 10-mile radius of the main study site, had a well-established membership of older adults who regularly participated in religious services and maintained ongoing relationships with their respective leadership such as pastors, ministers, and deacons (Fig 1).

### Recruitment

The recruitment process for this study was impacted by the COVID-19 restrictions and lockdown measures in effect during the study period (2019–2021). These restrictions required modifications to the recruitment approach, emphasizing virtual communication and online workshops to effectively engage and enroll participants. These adaptations prioritized the safety and well-being of all individuals involved, while strictly adhering to public health guidelines. Inspite of these challenges, the study’s lead community faculty (CF), who also serves as the head pastor of a participating Christian FBO, took the initiative to reach out to 15 head pastors (HP) and 25 influential Christian faith-based leaders (CFL) to encourage their participation in the research project. Convenient sampling was utilized to recruit the 17 Christian FBOs with no exclusions based on denomination or congregation size.

To select CHAs, potential candidates were conveniently sampled from the 17 partner Christian FBOs based on recommendations from individual HPs and CFLs. These trusted parishioners expressed interest and voluntarily signed up to become CHAs and were selected based on specific criteria including 1) age 18 years and older, 2) AA parishioner from a registered partner FBO, 3) attendance at a 3-day workshop training, 4) availability of at least 3 hours per week for study activities, 5) strong commitment to assisting older adults with chronic illness management and COVID-19 risk reduction, 6) familiarity with AA community, and 7) effective communication skills in both English and the preferred language of the older adults. It is important to emphasize that the selection process also offered an opportunity for HPs and FLs to participate as CHAs, provided they met the established inclusion criteria. The inclusion of HPs and FLs as CHAs aimed to leverage their influential leadership roles within their respective FBOs to bolster the implementation of the intervention and facilitate engagement with our target population.

The involvement of Christian FBOs played a crucial role in facilitating access to potential study participants, specifically targeting 250 individuals who were either 65 years and older or 55 years and older with at least one chronic health condition. The age criteria was chosen based on reported morbidity and mortality profiles which indicated increased vulnerability in these groups, especially in the context of the COVID-19 pandemic [36]. This decision was made to ensure the study would have the maximum impact on those at the highest risk [37]. Individuals residing in care facilities were excluded due to the higher risk of COVID-19 transmission in such environments [38, 39], coupled with access restrictions during the study period [40]. Additionally, participants with cognitive deficits, as identified using the short version of the mini-mental state examination instrument, were excluded to ensure that all participants could adequately comprehend and engage with the intervention materials and activities.

### Data collection

Data regarding participant and CHA sociodemographic characteristics, chronic health conditions, and health status were gathered through surveys. Participants completed the study surveys using different methods, including Uniform Resource Locator (URL), participating in a telephone interview, or undergoing an interview administered by the CHA. In addition to survey data, acceptance and completion rates throughout the intervention were assessed. To gain further insight into the perspectives of the FBO leaders and CHAs regarding the project and its implementation process, a combination of four informal and ten semi-structured in-depth interviews were conducted to complete the surveys. These interviews aimed to provide a deeper understanding of the viewpoints held by FBO leaders and CHAs. As part of the data-gathering process, CHAs and older adult participants worked collaboratively to identify and prioritize significant barriers and facilitators to implementing the intervention within their church or community setting.

### Analysis plan

Data analysis was conducted using descriptive statistics for the quantitative data and thematic analysis for the qualitative data. CFIR, a useful tool for assessing potential barriers and facilitators in implementing healthcare interventions, was utilized to guide the analysis of the implementation process and identify areas for improvement. This tool provides a practical, theory-based guide to tailor implementation strategies and adaptations based on these factors and explain the outcomes of the implementation process [41]. The project team also documented any adaptations made to the intervention based on feedback from the CHAs and older adults. The participants’ identified facilitators and barriers were grouped and reviewed before being compiled. The quantitative data were analyzed using SPSS (version 25). To produce a narrative report based on the CFIR domains, qualitative data were organized according to the CFIR constructs and sub-constructs.

### Implementation strategies

**Fig 2:**
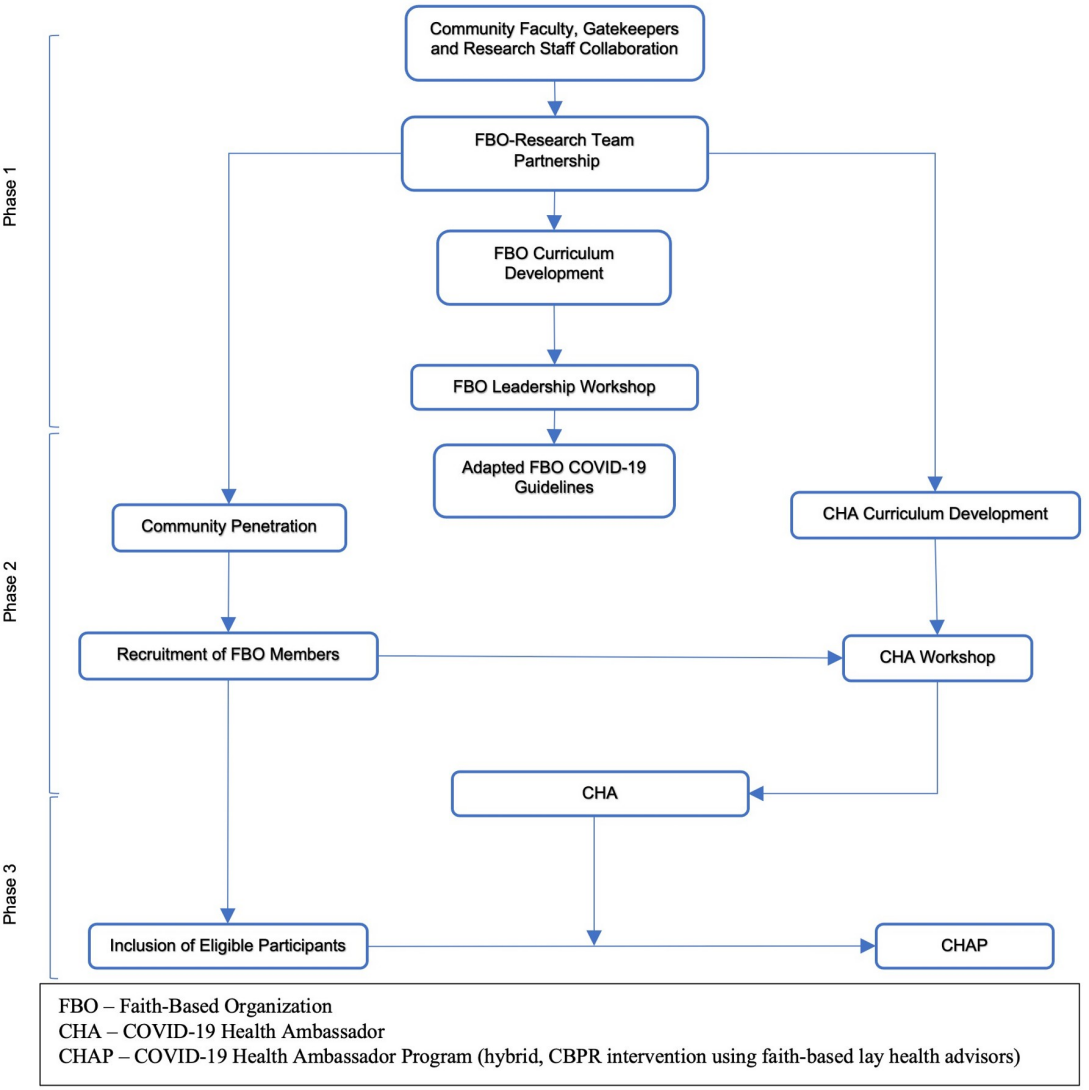
Conceptual model of a hybrid community-partnered intervention project using faith based lay health advisors.

Our project employed innovative implementation strategies by leveraging the trust and expertise of all stakeholders. These formed the basis of the project’s conceptual model (Fig 2) and broadly described below.

#### Community strategizing

A six-step strategy was developed to forge strong relationships with the FBO leadership and adapt COVID-19 public health guidelines for their respective denominations. This approach involved recruiting leaders, creating a specialized curriculum, and conducting a 3-day virtual training workshop to empower them in addressing COVID-19-related concerns within their communities. FBO leaders were responsible for implementing infection prevention measures, carrying out routine environmental assessments, and ensuring protocol compliance. Additionally, they participated in community outreach through the university’s weekly public radio show that promoted the CHAP’s goals and discussed culturally sensitive topics during the pandemic. These included, 1) ‘Managing COVID-19 Grief in Our Community’, 2) ‘Mental Health and Social Isolation among African Americans’, 3) ‘How to Mitigate Vaccine Barriers in our Communities’, 4) ‘How ‘Your Health Is Connected to Your Faith’, and 5) ‘Addressing Vaccine Hesitancy among the Youth; The Role of the Church’. FBOs supported the project by recruiting lay health advisors, coordinating communication between advisors and participants, and facilitating participant involvement.

#### Community building and sustainability

The individual-level collaborations established with FBO leadership enabled the development of a tailored CHA curriculum and the dissemination of the CHA workshop/training program. The courses within the workshop covered CHA roles, COVID-19 knowledge, testing and vaccination concerns, risk prevention strategies, social determinants, chronic disease management, available community resources, and specialized care delivery (Table 1). The 3-day virtual workshop was facilitated by FBOs, research/academic staff, healthcare providers, and community faculty, with recorded sessions available to those who missed the live event and for CHAs who needed refresher training. CHAs received iPads and were trained in confidentiality and data security.

**Table 1:** List of topics and presenters as used in training guideline for CHAs.

Topics Covered	Presenter
Functions and expectations CHAs	Research Staff/Church Leader
Medical & Research Mistrust	Public Health Specialist
COVID-19 testing and vaccination in community settings	Physician 1
COVID-19 vaccine equity	Physician 2
Mental health and COVID-19 related trauma	Mental Health Nurse
Chronic disease management (with and without COVID-19 diagnosis)	Physician
Telehealth and online services	Physician 3
How to be a church-based health advisor	Public Health Specialist /Church Leader 1
Motivational interviewing in the church	Church Leader 2
How to communicate with parishioners on their medications	Pharmacist 1
Alternative treatments to COVID-19	Pharmacist 1
Linking community resources to community needs	Community Faculty
Health tracking and data collection	Research Staff
Immunization	Public Health Nurse

#### Individualized management of care

The project team collaborated to create culturally appropriate intervention materials and trained CHAs to support participants via phone calls and videoconferencing remotely. The three-month intervention included five broad activities: 1) completion of pre- and post-intervention surveys, 2) attendance at six bi-weekly online/hybrid meetings, 3) participation in individualized checkups/visits, 4) project support and feedback, and 5) participation in a post-study conference/gala. The team coordinated with FBOs to align schedules and ensure CHA availability. CHAs helped older adults develop personalized action plans for managing chronic health conditions, addressing medication management, healthy eating, and physical activities.

#### Ethics statement

All participants provided written, informed consent before participating, and the study protocol was approved by the ethical committee of the Charles R. Drew University of Medicine and Science Institutional Review Board (IRB). Authors had no access to information that could identify individual participants during or after data collection.

## Results

The intervention engaged 17 AA FBOs in South Los Angeles, with additional churches in Southern California’s Inland Empire, Antelope Valley, and Victorville (n = 3). On a daily average, 47 CHAs from these FBOs attended the 3-day workshop, and 19 CHAs actively participated in the recruitment and support of participants. Each CHA aimed to recruit 10 participants, resulting in a 22% rejection rate, as 2 out of 10 parishioners averagely declined the 19 CHAs’ invitations to participate in the study’s baseline data collection. Consequently, out of the 152 participants who enrolled in the study, 110 participants completed the intervention, yielding a 72% completion rate.

The majority of participants (70%) were female, with a mean age of 69 (SD: 9), and 22% were aged ≥75 years as detailed in Table 2. Approximately 14% did not complete high school, 33% lived alone, and 98% had health insurance. Over 35% reported poor or fair physical health, and participants had an average of two chronic health conditions. The most prevalent comorbidities were hypertension (59%), COPD or asthma (24%), diabetes mellitus (22%), and heart disease (11%).

**Table 2:** Sociodemographic characteristics of Participants and CHAs.

Sociodemographic Characteristics	Participants Targeted for CHAP (Older African American Parishioners), N = 152, N (%)	CHAs, N = 19, N (%)
**Gender**		
Male	46 (30)	3 (16)
Female	106 (70)	16 (84)
**Age**		
40–54	2 (1)	1(5)
55–64	48 (32)	7 (37)
65–74	68 (45)	10 (53)
75 and older	34 (22)	1 (5)
**Education**		
No High School Diploma	20 (13)	0 (0)
High School Diploma	41 (27)	2 (27)
Some college/graduate	91 (60)	17 (73)

**Fig 3:**
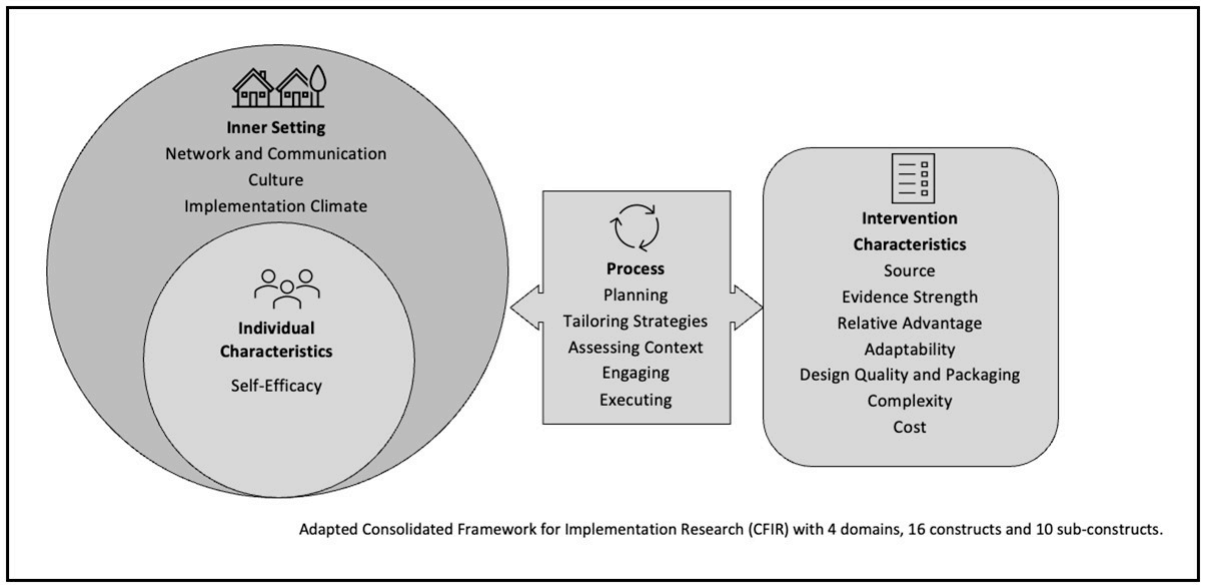
Adapted consolidated framework.

The implementation process was evaluated through a combination of informal interviews with FBO leadership, CHAs and participant survey items. Based on CFIR reporting guidelines, the findings highlighted the intervention characteristics (seven constructs), inner setting (three constructs, eight subconstructs), and the process (five constructs, two sub-constructs) as the most frequently addressed components of the CFIR, while individual characteristics (one construct) received comparatively less attention in the data. This is likely due to the intervention’s focus on the FBOs and the CHAs collectively rather than individually. The influential components of the CFIR that emerged from the data are illustrated in Fig 3.

### CFIR-related intervention characteristics

#### a) Source

CHAP emerged as a tailored solution based on the community’s expressed needs during the COVID-19 pandemic. This solution was the result of thoughtful initial deliberations between the research team’s community faculty and AA church/community leaders. Instead of being perceived as an external imposition, it was recognized as a grassroots response tailored to address specific chronic disease management challenges. The integration of CHAP within the AA faith-based framework and its alignment with prior relationships and community values significantly boosted its credibility and trustworthiness. Gathering stakeholder input and assessments throughout the project development process via virtual interviews, focus group discussions, and questionnaires was instrumental in understanding the holistic sentiment of each involved stakeholder.

#### b) Evidence strength

The project utilized a CBPR approach, considered a high-quality study design in the field of public health research [42]. The project’s use of faith-based health advisors aligns with evidence supporting this approach’s effectiveness in closing unmet needs gaps and decreasing health disparities for minority communities, including AAs [20, 21, 43–47]. The CDC "scaling up operation" recommends that faith-based organizations (FBOs) establish and maintain communication with local authorities and work closely with health organizations to implement COVID-19 guidelines effectively [22]. Telehealth methods had been suggested as an innovative approach to delivering healthcare services to vulnerable populations, such as AAs, who experience disparities in accessing and maintaining healthcare services [48–50]. AA churches have historically supported their families economically, spiritually, socially, and culturally through programs that are unmatched by other social institutions [20].

#### c) Relative advantage

In analyzing CHAP’s integration of virtual technology versus conventional in-person healthcare delivery mechanisms, multiple advantages became evident. CHAs highlighted that the virtual approach offered improved access to primary care providers, diminished barriers to receiving support, and enabled more consistent communication with participants. This virtual approach paved the way for delivering personalized and timely assistance, a noteworthy reduction in in-person health provider and community visits amidst the pandemic, and increased flexibility in scheduling provider appointments. This juxtaposition underscores the potential of the virtual system to address some of the shortcomings inherent in the conventional healthcare delivery model, especially during times of public health crises.

#### d) Adaptability

The COVID-19 pandemic greatly impacted chronic disease management for underserved older adults, especially when physical distancing measures significantly limited in-person visits. Initially virtual means of interaction used but it encountered technological barriers and challenges in building rapport. In response, the project team adapted to a hybrid model, model that blended both in-person and virtual check-ins, allowing for personalized care and improved communication.

#### e) Design quality and packaging

The research project ensured design quality by providing comprehensive training to FBO leaders and CHAs through a 3-day virtual workshop. FBO leaders received computers, while CHAs were given iPads, resource manuals, and biweekly discussion topics with detailed links to data and resource materials. Continuous feedback and response mechanisms were implemented through weekly meetings with research staff.

#### f) Complexity

The program’s nine-month span brought forth intrinsic complexities, including the mandatory bi-weekly meetings and maintaining regular communication between CHAs and older adults. The utilization of multiple communication methods- ranging from videoconferencing and phone calls to direct in-person sessions–added further depth to the complexity, especially in ensuring consistent and effective engagement. Ensuring the reliability of internet access and technology availability posed another set of challenges. However, the team’s approach in providing continuous technical support and troubleshooting helped navigate through these technological intricacies. Managing these overlapping layers of complexity was also essential for further accurately capturing supplemental nutritional, physical activity, and food insecurity data in pre- and post-intervention surveys.

#### a) Cost

The CHAP initiative demanded meticulous financial planning. Delving into its cost structure, initial expenditures were directed towards infrastructure: technology pivotal for virtual communication, training modules for FBO leaders and CHAs, and essential materials aiding their roles. While the expert team of workshop resource persons (Table 3) volunteered their training expertise sans compensation, there were indispensable costs. These included compensations for survey and evaluation completions, salaries for three key research team members, and funds earmarked for an end-of-project conference—envisioned as a space for knowledge exchange, results dissemination, and stakeholder dialogue. Despite these significant costs, the potential for long-term savings became apparent with fewer in-person visits and augmented accessibility to primary care via telehealth.

**Table 3:** Recruitment and completion rates of CHAs and participants.

	Cohort One	Cohort Two	Total
**Faith-Based Organizations**			
Participating Churches	8	9	17
**Faith-Based COVID-19 Ambassadors**			
Prospective Number of COVID-19 Ambassadors (three per church)	24	27	51
Trained COVID-19 Ambassadors	24	23	47
Active COVID-19 Ambassadors during Intervention	14	5	19
Participation rate of Trained COVID-19 Ambassadors (trained/expected number)	100%	85%	92%
Participation rate of Active COVID-19 Ambassadors (active/trained)	58%	22%	40%
**African American Older Adult Parishioners (Participants targeted for CHAP)**			
Number of prospective participants (10 participants from each active ambassador)	140	50	190
Number of recruited participants pre-CHAP (completed baseline survey)	118	34	152
Number of participants who completed CHAP	90	20	110
Percentage of participants who completed CHAP (participants who completed CHAP/participants recruited pre-CHAP)	76%	59%	72%

### CFIR-related inner settings

The research team implemented a comprehensive communication plan promoting regular stakeholder engagement, including virtual weekly check-ins between research staff and CHAs. The approach facilitated collaboration and innovation by fostering strong relationships between CHAs, research staff and the older adults they served. It also promoted peer-support groups and team-building activities among CHAs, which proved crucial during the COVID-19 pandemic.

#### a) Network and communications

Within each participating FBO, a well-established system of communication became a linchpin for the intervention’s success. The faith-based organizations’ inherent structure facilitated seamless interactions between participants and the research team. The existing rapport and trust within the community, accentuated by the CHAs’ role, ensured that the communication was not just frequent but also meaningful. This robust network, built on shared values and goals, played a pivotal role in surmounting the challenges posed by the complexity of the intervention. It demonstrated the crucial role of internal communication systems in supporting and enhancing the objectives of external interventions like CHAP.

#### b) Culture

The use of CHAs from the local churches further ensured that the project was culturally appropriate and responsive to the community’s needs. Several specific examples of the culture construct can be highlighted to demonstrate how it was addressed and integrated throughout this project’s implementation process. Some included:

*Cultural beliefs about health and illness*. The project addressed community preferences for traditional or alternative medicine by training CHAs to incorporate these beliefs into evidence-based self-management strategies.

*"As a CHA*, *I encountered an older adult who relied on herbal remedies to manage their diabetes*. *I worked with them to ensure these remedies were used alongside conventional treatments*, *resulting in better blood sugar control*.*"*

(FBO#1,CHA#2)

*Dietary practices and preferences*. The research team ensured that culturally relevant nutritional advice was provided, considering specific dietary practices and preferences within the AA community.

*"One of my older adults loved soul food*, *so I helped them find ways to prepare their favorite dishes using healthier ingredients and cooking methods*, *making their meals both culturally satisfying and beneficial to their health*.*"*

(FBO#2,CHA#1)

*Language and communication*. CHAs were selected based on their familiarity with the cultural context and communication styles of AA older adults, and educational materials were modified to be culturally appropriate.

*"We made sure our educational materials spoke to our community*, *using analogies and references that our older adults could easily relate to*. *This approach made complex health concepts more accessible and understandable*.*"*

(FBO#4,CHA#2)

*Family and community dynamics*. CHAs were encouraged to engage with the older adults, their families, and community networks, fostering a supportive environment for self-management strategies.

*‘Most of the people I recruited were my family–my husband and sisters*. *Going through this process*, *I was able to find out about everyone’s health issues that I never knew they dealt with in the past*.*’*

(FBO#1,CHA#1)

*Traditional community gatherings*. The research team leveraged traditional AA community gatherings as opportunities to raise awareness about CHAP and actively engage with the community, showing respect for the community’s culture and building trust with older adults and their families.

*"I used our weekly dinner and bingo sessions to talk with community members about the health ambassador program*. *We shared stories*, *addressed concerns*, *and offered support in a relaxed*, *familiar environment*. *It helped us build trust and connect with the community on a deeper level*.*"*

(FBO#5,CHA#3)

#### c) Implementation climate

Creating the right implementation climate for the CHA intervention required a sense of shared commitment, necessary resources, training, and clear goals. Issues stemming from ambiguity, including confusion about objectives, roles, and expected outcomes, were addressed through additional meetings and workshops. Also by the development of detailed guidelines, the establishment of more explicit performance metrics, and the implementation of regular check-ins with CHAs and community partners. These efforts helped with maintaining clarity, resolve emerging issues, and reinforce the shared commitment to the program’s goals.

*Tension for change*. This awareness of existing health disparities within our target communities created a sense of urgency and motivation for change among the research team, CHAs, and community partners, fostering a conducive environment for implementing the CHA intervention. The lack of support perceived by AA older adults, coupled with heightened concern, misinformation, disinformation, confusion about the messaging and pandemic-related risks increased this tension for change, as stakeholders recognized the need for additional support and resources.
As the CHA intervention was implemented, the research team collected success stories from older adults who had benefitted from the support provided by the CHAs. Sharing these success stories with the broader community, including those who were initially resistant to the program, helped demonstrate the intervention’s positive impact and increased the tension for change by showcasing the program’s benefits.
*Compatibility*. To address the specific needs of each participant, the project allowed CHAs to customize the delivery of interventions based on the choices and circumstances of each older adult (*Kangovi et al*., *2014*). Depending on the participant’s comfort level and accessibility, CHAs could choose between selected in-person visits, phone conversations, or virtual meetings. This adaptability boosted the program’s compatibility with the diverse demands of the older adults and contributed to sustaining participation throughout the intervention.
*Relative priority*. Non-participating local church leaders and FBOs collaborated with the project team, actively referring older adults in need of CHA support and participating in the evaluation and improvement of the intervention.

### CFIR-related individual characteristics

#### Self-efficacy

The project equipped CHAs with the knowledge, skills, and resources to provide tailored support, broadly categorizing the approach into training, social support, and peer modeling.

Training: CHAs were trained to employ goal-setting, problem-solving, and self-monitoring techniques/approaches to assist older persons in building confidence in their abilities to manage chronic diseases and stick to treatment programs.

*Peer modeling*. The CHAs, who shared comparable cultural and socioeconomic backgrounds with the older persons they supported, functioned as role models by displaying good self-management skills and highlighting success stories from their communities. Peer modeling played a crucial impact in boosting older individuals’ self-efficacy.
*Social support*. The CHAs also gave older persons with social support, boosting their self-efficacy by establishing a sense of belonging and connection. This assistance comprised emotional encouragement, educational support, and practical solutions in managing their chronic diseases. CHAs provided reassurance, answered concerns, and assisted with chores such as organizing medical visits and navigating the healthcare system.

### CFIR-related process

#### a) Planning

The project team began by clearly outlining the objectives and goals of the intervention, such as improving chronic disease management among participants and increasing their engagement in health-promoting behaviors. Next, the team conducted a comprehensive assessment of the available resources, including human and technological resources, to support intervention implementation. For instance, we identified local health professionals within the churches who could serve as CHAs and ensured access to necessary technological tools. Following the resource assessment, the project team developed a detailed implementation timeline, which included milestones and deadlines for each phase of the project (Table 4). Lastly, acknowledging the potential for unforeseen challenges, particularly in light of the uncertainties posed by the COVID-19 pandemic, the project team developed contingency plans to address potential barriers. For instance, we prepared alternative training methods and modes of communication to accommodate potential lockdowns or changes in public health guidelines.

**Table 4:** Project timeline.

Activity	Months					
	1–3	4–6	7–9	10–14	15–19	20–24
Recruitment of FBOs	x					
FBO curriculum development and workshops	x					
Recruitment and training of FBO lay health advisors	x	x				
Recruitment of participants		x	x			
Collection of baseline data from participants			x			
Implementation of CHAP				x	x	
Collection of post-CHAP data from participants and CHAs						x

#### b) Tailoring strategies

The need for tailored strategies became evident through initial interactions with different FBOs. For instance, while one church had a strong tradition of holding weekly health seminars, another had a robust system of home visits by church elders. Capitalizing on these pre-existing structures, for the former, we integrated our health messages into their weekly seminars, making them more resonant with the congregation’s expectations. For the latter, we incorporated health check-ins during home visits, leveraging the trust and rapport that the elders had built over time. Such customizations ensured that the interventions were not seen as external impositions but seamlessly integrated into the fabric of the FBOs’ practices.

#### c) Assessing context

The contextual assessment delved deeply into the dynamics of the community, unearthing some significant barriers to healthcare access. A recurring theme, highlighted by many participants, was a deep-seated medical mistrust, rooted in historical injustices and discriminatory practices experienced by the African American community [30, 51]. By acknowledging this mistrust, we ensured that our strategies were not just about disseminating health information but also about building trust and rapport. Efforts were made to involve trusted FBO figures, like head pastors or respected leaders/elders/administrators, in the intervention. Their involvement acted as a bridge, ameliorating some of the mistrust and facilitating more open conversations about health and disease management.

#### d) Engaging

Integral to the project’s success was the proactive engagement of stakeholders at every phase. Beyond the typical participants, a concerted effort was made to involve local health professionals, faith leaders, and community/FBO influencers. Out of the initial 17 FBOs representing the study’s first cohort, 10 actively participated alongside their respective leaders. The participation commenced with each FBO leader receiving an invitation via email containing information regarding the study. Once we received their interest in participating, the research team met with these leaders to discuss expectations and the research plan. FBO leaders were crucial in recruiting potential volunteers as CHAs drawing upon their experience, interest, and capacity to work with older adults in their congregation. FBOs held virtual meetings to convey the project’s goals and objectives to interested volunteers and provided them with eligibility criteria details. They also used various communication channels such as bulletins, newsletters, and announcements during the few in-person services to disseminate information about volunteer opportunities.

*Opinion leaders (Formally appointed implementation leaders)*. As internal implementation leaders, FBO leaders faced competing priorities and interests, making it challenging to consistently support the project. To address this, the research team implemented strategies such as flexible scheduling, frequent communication, and progress updates. The research team maintained their support and involvement by accommodating the FBO leaders’ busy schedules.
*Champions*. CHAs faced challenges balancing responsibilities and staying updated with relevant information. The research team supported CHAs through refresher training, equipped them with Apple iPads, offering recognition, conducting weekly check-ins, and maintaining ongoing communication. By addressing these challenges and offering flexible scheduling, the team created an environment for CHAs to thrive.

#### e) Executing

To enhance CHA recruitment efforts by leaders of various FBOs, the research team used a combination of strategies and procedures to ensure we attracted and retained a dedicated, competent and diverse group of individuals. These strategies and procedures included:

*External collaborations*. The research team partnered with local community organizations, such as African American Community Empowerment Council (AACEC), to identify potential CHAs within their target population. This approach facilitated the recruitment of individuals who were already active in their communities and had established connections with the older adults they would serve.
*Word-of-mouth referrals*. The research team encouraged existing CHAs and other community members involved in the project to refer potential candidates from their personal and professional networks. This strategy helped with identifying individuals passionate about helping others and had the interpersonal skills necessary to succeed as a CHA.
*Social media*. The research team utilized various advertising channels, such as our FBOs’ social media platforms and radio announcements, to broaden their reach and inform a wider audience about the opportunity to become a CHA. This strategy increased the visibility of the project and attracted a diverse pool of candidates.

## Discussion

Our findings from 17 AA churches in South Los Angeles revealed a high project completion rate of 72% among participants. This success can be attributed to the implementation of a culturally sensitive, contextually relevant, and responsive approach achieved through CBPR. This approach, which involved partnering with community churches, likely increased participants’ trust and engagement with the intervention. [42, 52, 53]. These findings align with existing literature on community-based health interventions targeting underserved populations [28, 29]. A study by Kangovi and colleagues (2014) observed significant improvements in chronic disease control and mental health outcomes with a community health worker intervention implemented in a low-income urban population and [54]. Similarly, Resnicow and colleagues (2006) found that a culturally tailored intervention was more effective in achieving behavior change among AA adults attempting to increase fruit and vegetable consumption [55].

We also observed a considerably higher (70%) number of female participants in this study. This gender disparity is consistent with findings from Kangovi and colleagues (2014) who observed a larger number of female participants in their study (69%) [54]. In AA communities, women often play vital roles in the social and spiritual life of their communities, with churches traditionally serving as the focal points [15]. Women tend to be more health-conscious and are more likely than men to participate in preventive health initiatives [56]. They are also more inclined to seek medical care, adhere to medical advice, and engage in health-promoting behaviors [57]. CHAP’s focus on underserved AA older adults with chronic health conditions is consistent with other research highlighting the need for targeted interventions in this population [58, 59]. For instance, Lorig and colleagues (2001) found that a self-management program targeting self-efficacy in patients with chronic diseases improved health status, self-management behaviors, and healthcare utilization [58].

This study also highlights the effectiveness of a hybrid approach combining virtual and in-person visits. Virtual technology improved access to care, reduced barriers, and offered increased flexibility in scheduling provider appointments, but faced challenges such as technological barriers and maintaining rapport between CHAs and participants [60, 61]. Adapting to the hybrid model provided personalized care and improved communication while addressing the limitations of CHAP’s initial approach [62]. This approach aligns with findings from several studies that have examined the benefits of hybrid interaction models in various populations and contexts. These studies have found that a hybrid telehealth model improved patient satisfaction, access to care, and health outcomes among older adults with multiple chronic health conditions [63–66]. Similarly, Hollander & Carr (2020) reported that telehealth and in-person visits enhanced patient engagement and facilitated timely care, particularly during the pandemic when access to healthcare was limited [62]. In another study, Greenhalgh and colleagues (2020) emphasized the importance of adaptability and a hybrid approach to ensure personalized, flexible patient care strategies during the pandemic [67]. Furthermore, Smith and colleagues (2020) highlighted the benefits of virtual communication in expanding access to healthcare and mitigating barriers faced by underserved populations, including older AA adults [60].

Clear communication and robust relationships were vital for the project’s success, as CHAs offered tailored support to older adults [60, 61]. The project’s cultural sensitivity enhanced its acceptability among the AA older adult population. A supportive environment for implementation was crucial, with effective communication addressing health disparities and risks associated with the pandemic such as misinformation and vaccine hesitancy [62, 67]. Tailoring the intervention delivery ensured compatibility and maintained participant engagement throughout the intervention [54]. The current project’s cooperation with local church leaders and FBOs aligns with Smith and colleagues (2014) (2020), who also found that engaging FBOs was crucial in recruiting community health advisors and ensuring the success of their intervention [60]. This collaboration reinforces the significance of community engagement and partnership in promoting the reach and impact of health initiatives.

### Limitations

The project took place during the height of the COVID-19 pandemic, which presented unique challenges to managing chronic health conditions for African American older adults, particularly those who were isolated or had limited access to healthcare. Several limitations should be considered when interpreting the findings of this study. The study focused exclusively on Christian FBOs, CHAs, and participants from predominantly AA FBOs. This limits the generalizability of the findings to individuals from other religious backgrounds, such as Islam, Hinduism, and Buddhism. Future research should include a more diverse range of religious groups to better understand the influence of religious diversity on health disparities and the effectiveness of faith-based interventions. Second, self-report measures were used for data collection, which may be subject to recall bias or social desirability bias. It is important to acknowledge that the COVID-19 restrictions and lockdown measures had an impact on the recruitment process, potentially influencing the reach and diversity of the study population. Finally, the study’s short-term nature may restrict the assessment of long-term outcomes of COVID-19. Despite these limitations, this study provides valuable insights into the implementation and impact of CHAP, contributing to the literature on addressing health disparities among underserved AA older adults during the COVID-19 pandemic.

## Conclusions

Overall, the findings from this study offer significant insights into the implementation process and highlight essential factors to be considered when designing interventions for similar contexts. These insights contribute valuable knowledge about the feasibility, acceptability, and impact of a hybrid (virtual/in-person) lay health advisor model during the pandemic. They can guide the development of future interventions in this domain. Our results are also consistent with existing research on the effectiveness of community-based health interventions for underserved populations. The high completion rate and improvements in chronic health condition management underscore the necessity of incorporating culturally sensitive, contextually relevant, and responsive strategies in healthcare interventions aimed at underserved African American older adults.

## Supporting information

S1 Dataset Minimal dataset containing primary data obtained from CHAP participants. (CSV)
